# Sulfur monoxide dimer chemistry as a possible source of polysulfur in the upper atmosphere of Venus

**DOI:** 10.1038/s41467-020-20451-2

**Published:** 2021-01-08

**Authors:** Joseph P. Pinto, Jiazheng Li, Franklin P. Mills, Emmanuel Marcq, Daria Evdokimova, Denis Belyaev, Yuk L. Yung

**Affiliations:** 1 University of North Carolina at Chapel Hill, Chapel Hill, NC USA; 2 Division of Geological and Planetary Science, California Institute of Technology, Pasadena, CA USA; 3 Australian National University, Canberra, ACT Australia; 4 Space Science Institute, Boulder, CO USA; 5 LATMOS/CNRS/Sorbonne Université/UVSQ, Paris, France; 6 Space Research Institute of the Russian Academy of Sciences (IKI), Moscow, Russia; 7 Jet Propulsion Laboratory, California Institute of Technology, Pasadena, CA USA

**Keywords:** Planetary science, Atmospheric chemistry

## Abstract

The abundance of SO dimers (SO)_2_ in the upper atmosphere of Venus and their implications for the enigmatic ultraviolet absorption has been investigated in several studies over the past few years. However, the photochemistry of sulfur species in the upper atmosphere of Venus is still not well understood and the identity of the missing ultraviolet absorber(s) remains unknown. Here we update an existing photochemical model of Venus’ upper atmosphere by including the photochemistry of SO dimers. Although the spectral absorption profile of SO dimers fits the unknown absorber, their abundance is found to be too low for them to contribute significantly to the absorption. It is more likely that their photolysis and/or reaction products could contribute more substantively. Reactions of SO dimers are found to be important sources of S_2_O, and possibly higher order S_n_O species and polysulfur, S_n_. All of these species absorb in the critical ultraviolet region and are expected to be found in both the aerosol and gas phase. indicating that in-situ high resolution aerosol mass spectrometry might be a useful technique for identifying the ultraviolet absorber on Venus.

## Abstract (web summary)

Photochemistry of sulfur species in the upper Venus atmosphere is not well understood and the identity of ultraviolet (UV) absorber(s) remain unknown. Here, the authors show that sulfur monoxide dimer chemistry is a possible source of polysulfur, which could be responsible for the UV absorption.

## Introduction

The photochemistry of sulfur species in the upper atmosphere of Venus is not well understood and is poorly quantified. The known parent molecules upwelling from the deep atmosphere are SO_2_ and OCS. Near and above the cloud tops, these molecules are photolyzed, leading to the production of S_n_ (*n* = 1–7), S_x_ (x = 8+), SO, SO_3_, and H_2_SO_4_^1^. Significant amounts of sulfur aerosol were predicted in the lower and middle cloud layers by ref. ^2^ and also ref. ^3^, which assumes a particle radius of 0.5 μm. Ref. ^4^ suggested that the dimers of sulfur monoxide (SO), (SO)_2_, formed from the self-recombination of SO, could contribute substantially to the ultraviolet (UV) absorption found on Venus. In addition, production of S_n_O could also occur, as suggested by ref. ^5^.

There are three main isomeric forms of the SO dimer. Reference^4^ concluded that SO recombination yields ~49% as *cis*-OSSO, ~49% as *trans*-OSSO isomers and no more than 2% as the trigonal isomer (S=SO_2_) which is the lowest energy isomer. Reference ^6^ considered only the formation of the trigonal form, photolysis of which leads to the formation of S and SO_2_. Reference^3^, based on photochemical modeling, suggested that the abundance of OSSO is too low by two orders of magnitude to explain the near UV absorption of Venus. Photolysis of SO dimer is expected to yield mainly two SO radicals^4^, but we will consider other possible reaction paths. Reference ^7^ found evidence for the formation of the *cis*-OSSO, *trans*-OSSO, and C1-S_2_O_2_ (cyclic OS(=O)S) dimers, along with several other isomeric forms in much lower abundance, following condensation into a solid matrix with N_2_ and subsequent irradiation. Production of S_2_ and SO_2_ was also observed, but the trigonal (S=SO_2_) dimer form was beneath detection in the observations of ref. ^7^. S_2_ was formed initially from dissociation of the ethylene episulfoxide used as source of SO; further irradiation at 365 nm depleted the 370 nm band, which they ascribed to destruction of syn-OSSO (*cis*-OSSO). This depletion occurred simultaneously with an increase in absorption in the 287 nm band of S_2_. Formation of S_2_ requires only 1 eV above that for dissociation of dimer back to SO and could account for the very strong absorption and characteristic vibrational structure seen at 287 nm in their experiments. We suggest that the photochemistry of (SO)_2_ could provide a significant source of S_2_ on Venus based on the results of ref. ^7^, leading ultimately to the production of polysulfur, a candidate for the unidentified UV absorber on Venus^1^ and possibly also to production of polysulfur oxides^5^.

In this work, we use a photochemical model to assess the contributions of the proposed dimer chemistry to the production of polysulfur. We find that the SO dimers are more likely to be important intermediaries in the formation of more complex S species that could be responsible for the UV absorption. Our model chemistry is also applied to interpret data from the European Space Agency’s Venus Express (VEx) mission that was recently processed and analyzed and to provide input to plans for future missions.

## Results

**Fig. 1:**
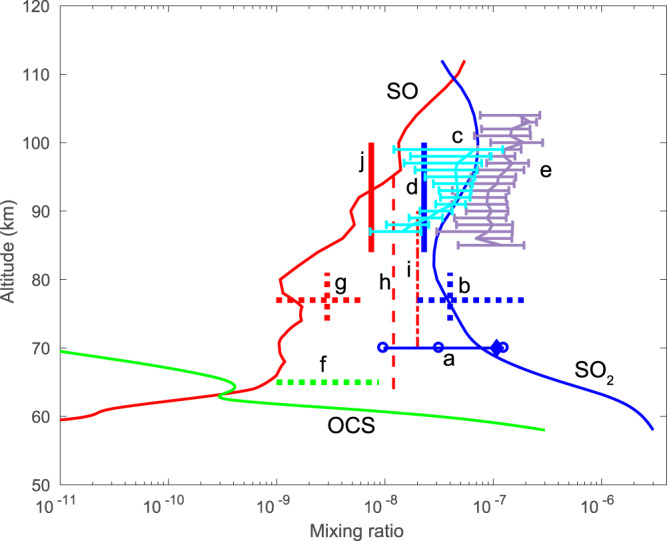
Modeled profiles of SO2, OCS, and SO. Comparison of modeled profiles of SO_2_, OCS and SO with observations for SO_2_ = 3.0 ppm and OCS = 0.3 ppm at 58 km, the lower boundary of the model. Model profiles are shown as thin solid lines: (blue) SO_2_, (green) OCS, (red) SO. Data sources: **a** SO_2_, interquartile range from 2006 through 2009, diamond shows mean of distribution, ref. ^10^; **b** SO_2_, ref. ^11^; **c** SO_2_, ref. ^34^ solar occultation with 1-σ error bars; **d** SO_2_, ref. ^12^; **e** SO_2_, ref. ^34^ stellar occultation with 1-σ error bars; **f** OCS, ref. ^35^; **g** SO, ref. ^11^; **h** SO, ref. ^14^; **i** SO, ref. ^13^; **j** SO, ref. ^12^.

Figure 1 presents the mixing ratios of SO_2_, OCS, and SO in the middle atmosphere using an SO_2_ mixing ratio of 3.0 ppm, which we have adopted for our standard model at the lower boundary (58 km), based on ref. ^8^ and 0.3 ppm for OCS, based on ref. ^9^. The profiles of the rates of the reactions in Supplementary Table 1 are shown in Figure Supplementary Fig. 1. Comparison of the model with several observational datasets at higher altitudes is also shown in Fig. 1. As can be seen (curve a), the model is in reasonable agreement with SO_2_ mixing ratios at 70 km during the first four years (2006 through 2009) of VEx measurements^10^. Calculated values are slightly higher than the interquartile range and the mean of VEx measurements within ±20° latitude; but the data include a large number of spikes, as indicated by the difference between the median and mean observations. As an indication of the model sensitivity to the choice of SO_2_ at the lower boundary, we also show the model-measurement comparison using an SO_2_ mixing ratio of 0.3 ppm at the lower boundary in Supplementary Fig. 2. This lower boundary condition could also be seen as reflecting in some ways the lower cloud top SO_2_ mixing ratios seen from the start of 2010 through 2014. Calculated values are within the interquartile range and are quite close to the mean of VEx measurements within ±20° latitude. As is the case for the earlier period in the record, the mean differs significantly from the median because of the existence of a large number of concentration spikes, which are roughly three orders of magnitude greater than median levels.

Modeled SO_2_ (thin solid blue line) is also consistent with Hubble Space Telescope (HST) data (dashes b) of ref. ^11^ and with the upper limit derived from ground-based submillimeter observations (dashes d) of ref. ^12^ from 85 to 100 km and is in reasonable agreement with Spectroscopy for Investigation of Characteristics of the Atmosphere of Venus/Solar Occultation at Infrared (SPICAV/SOIR) measurements from 90 to 100 km (curve c) of ref. ^13^ collected from 2006 through 2014. Note that the upper level SO_2_ mixing ratios (curve c) obtained by ref. ^13^ from 2006 through 2009 tend to be higher than those from 2010 through 2014 in a manner similar to SO_2_ observed at 70 km by ref. ^10^. Both ground-based submillimeter spectroscopy^12^ and solar and stellar occultations^13^ with one standard deviation (curve c and e) show higher SO_2_ mixing ratio at high altitudes (z > ~85 km) compared to lower altitudes. There are two possible sources of SO_2_ to be considered at these altitudes, meteorite ablation and photolysis of H_2_SO_4_. We have included ablation of meteoritic material, which is a very minor source of S to the upper atmosphere of Venus based on current estimates of meteoric input^14^ and S content^15^. The calculated profile of H_2_SO_4_ is within limits obtained by ref. ^16^. Photolysis of H_2_SO_4_ is also included based on calculations of its absorption spectrum by ref. ^17^, which provided evidence for the existence of a long-wavelength tail that substantially increases the photolysis rate of H_2_SO_4_ in the current model.

The reasons for the differences between observed and calculated SO_2_ and OCS for some datasets shown in Fig. 1 are not entirely clear, but some of the applicable datasets share the common feature that they were obtained on relatively short time scales. The SO_2_ record at 70 km exhibits dramatic spatial and temporal variability with SO_2_ mixing ratios in the equatorial region spanning three orders of magnitude from ppb to ppm levels. Data for some datasets could have been obtained during the brief periods when vertical transport was greatly enhanced compared to the mean values used here. Examining variability in transport as a source of variability in mixing ratios of SO_2_ and OCS is beyond the scope of this paper and is best accomplished using a multidimensional model incorporating radiative, chemical and dynamical feedbacks.

Reasonably good agreement of modeled SO with Hubble Space Telescope observations of ref. ^11^ and the mean SO abundance determined by submillimeter observations of ref. ^12^ at higher altitudes is found using a model with 3.0 ppm SO_2_ at the lower boundary. Larger model-observation differences are found for the SO mixing ratios observed by ref. ^18^ and ref. ^19^ beneath an altitude of about 80 km. Reference ^18^ concluded that IUE data were best fit with an SO mixing ratio of 20 ± 10 ppb above ~70 km with no SO beneath that altitude; ref. ^19^ derived a best fit to their data with a constant mixing ratio of SO of 12 ± 5 ppb for z ≥ 64 km and falling off sharply beneath 64 km. As can be seen from Fig. 1, our calculated mixing ratio of SO is 0.14 ppb at 64 km, increasing with height. As can also be seen from Fig. 1, our calculated mixing ratio of SO is ~1 ppb at 70 km.

The SO_2_ profiles shown in Supplementary Fig. 2 (solid blue line) calculated using 0.3 ppm at the lower boundary consistently underpredict the higher altitude observations. As seen in Supplementary Fig. 2, a mixing ratio of 0.3 ppm for SO_2_ applied at the lower boundary leads to an SO mixing ratio that is slightly too low compared to observations (dashes g, h, i, j). However, much of the disagreement may simply be due to the sparse nature of observations of SO that are not able to capture the extent of spatial and temporal variability as was possible for SO_2_.

**Fig. 2:**
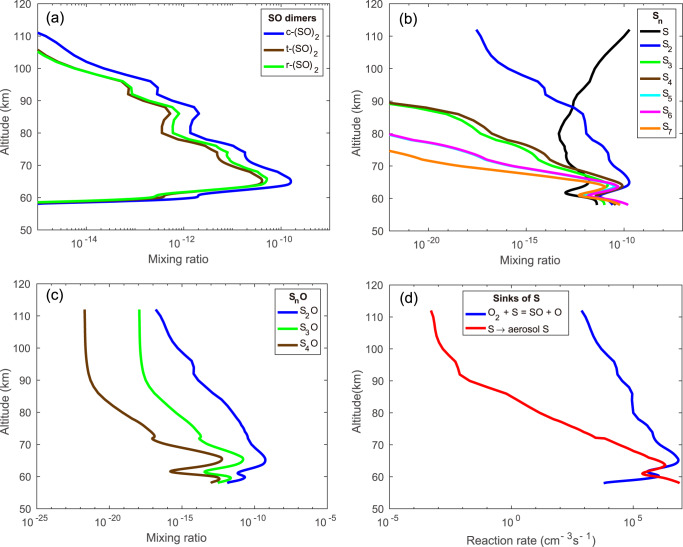
Model results. **a** Modeled profiles of *cis*-(SO)_2_, *trans*-(SO)_2_, and trigonal-(SO)_2_. **b** Modeled profiles of S_n_ species. **c** Modeled profiles of S_2_O, S_3_O and S_4_O. **d** Comparison of production of aerosol S versus recycling of S back to SO.

The mixing ratios of the three isomers of (SO)_2_, c-(SO)_2_, t-(SO)_2_, and r-(SO)_2_, are shown in Fig. 2a. Our values are consistent with the model of ref. ^3^ but are far lower than those calculated by ref. ^4^, particularly in the crucial layer beneath 70 km. The reason is that ref. ^4^ fixed the mixing ratio of SO to 12 ppb at 64 km based on the model of ref. ^19^ while decreasing it to 3 ppb at 70 ppb based on the microwave observations of ref. ^12^ and then increasing it to 150 ppb at 96 km based on ref. ^20^. The altitude profile of SO derived by ref. ^19^ should be reevaluated in the context of the shape of the modeled profile, which shows an increase in the mixing ratio of SO with height. This is a more likely shape for the SO mixing ratio profile based on measurements of ref. ^11^, ref. ^12^ and current understanding of photochemistry. It must also be kept in mind that conditions on Venus are highly spatially and temporally variable, so short-term measurements might not represent conditions at other times. The mixing ratios of S_n_ (*n* = 1–7) are shown in Fig. 2b and S_n_O (*n* = 2–4) are shown in Fig. 2c.

There is competition between production of reduced aerosol and recycling of oxidized S. It is clear from Fig. 2d, that the reaction, S + O_2_ → SO + O, is the primary sink for S throughout the modeling domain, except in a few thin layers at about 64 km and below. This demonstrates the difficulty of producing S_n_ via S atoms, which are rapidly converted to SO in the presence of O_2_. In our current model, production of S_2_ does not directly involve S atoms, as it is derived from alternative paths involving SO dimer photochemistry and as seen below from catalytic cycles involving Cl, thus bypassing the rapid recycling of S to SO by O_2_. Sensitivity studies carried out by varying the rate coefficients in Supplementary Table 1 uniformly upward and downward by a factor of ten indicate that species concentrations are typically within a factor of two of their values shown in Supplementary Table 1.

Cl has been shown to facilitate the stability of CO^21–23^ Cl also exerts strong influence on the production of S_x_. The column production rate of aerosol (on an S basis) is 8.3 × 10^11^ cm^−2^ s^−1^ in our standard model (SO_2_ = 3.0 ppm, HCl = 0.4 ppm) but it is reduced to 6.4 × 10^11^cm^−2^ s^−1^ for HCl = 0.2 ppm. At the lower SO_2_ mixing ratio (SO_2_ = 0.3 ppm) used at 58 km, the results are even more dramatic. Column production rates of aerosol drop from 2.9 × 10^11^ cm^−2^ s^−1^ to 1.1 × 10^11^ cm^−2^ s^−1^ for a decrease in HCl mixing ratio from 0.4 to 0.2 ppm. Although changes in major species such as SO_2_ associated with changes in HCl are relatively minor, order of magnitude changes in S and Cl-S species are seen. This strong sensitivity results in part from Cl reactions depleting O_2_, the major sink for S, through recombination of CO_2_. This is similar to what was found for production of S_2_ via chlorosulfanes^24^.

**Fig. 3:**
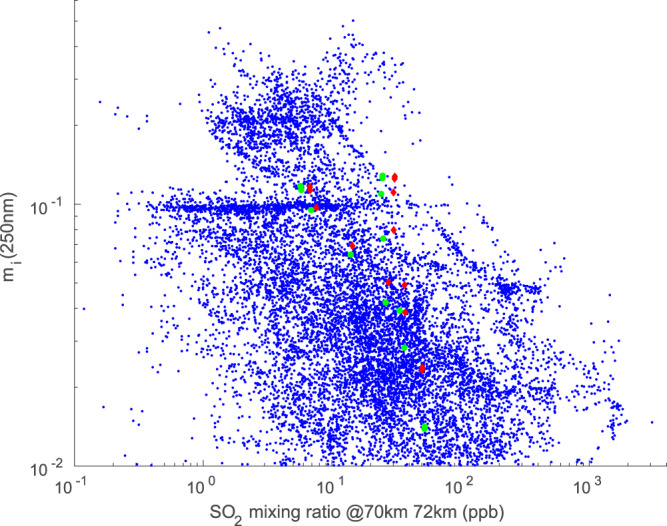
Correlation between S_x_ and SO_2_. Scatterplot showing imaginary index of refraction at 250 nm versus SO_2_ at 70 km from Venus Express^10^ (blue dots) compared to the scatterplot of S_x_ versus SO_2_ at 70 km (red diamonds) and 72 km (green dots) obtained from our model. The mixing ratios of S_x_ in our model are multiplied by 2 × 10^5^ in order to compare with the imaginary index of refraction derived from ref. ^10^.

There are a number of intriguing clues linking SO_2_ photochemistry to the unknown absorber. As shown in Fig. 2d, the maximum production rate of absorbing aerosol in our model occurs in the lower half of the upper cloud deck, but noticeable depletion of SO_2_ occurs only at higher altitudes. Our results are consistent with VEx observations showing an inverse relation between SO_2_ at 70 km and UV absorption at 250 nm^10^, as shown in Fig. 3. The data points in Fig. 3 are obtained from the time steps towards equilibrium when running our model and are meant to provide an indication of the adequacy of the model’s production rate of absorbing aerosol. The density of S_x_ is found to be negatively correlated with the local SO_2_ mixing ratio, which is consistent with the observations of ref. ^10^. However, as noted by ref. ^25^, the lifetime of the UV absorber is much longer than that of SO_2_, and as a result, the ratio of their abundances is strongly affected by transient atmospheric dynamics, in particular convective activity. Upward transport in the ascending branch of the Hadley Cell could bring absorber upward from where it is formed in the lower half of the upper cloud deck. Events such as these would reduce the strength of any correlation between SO_2_ and S_x_. Since our one-dimensional model is meant to simulate mean conditions at low latitudes, it cannot encompass the full range of conditions sampled by *Venus Express*. It therefore cannot simulate the behavior of SO_2_ and the UV absorber undergoing poleward transport; a detailed simulation of their latitudinal behavior requires a two- or three-dimensional model.

## Discussion

The SO dimers are more likely to be important intermediaries in the formation of more complex S species that could be responsible for the UV absorption, rather than being the UV absorbers themselves. Cl is an important modulator of important chemical processes maintaining the stability of CO_2_ and the production of condensable sulfur species. These species include S_x_ and S_n_, which have been the subject of several studies (see e.g. ref. ^24^ and references therein), and possibly S_n_O^5^. In our current model, which only includes condensational loss to existing particles and does not consider new particle formation, S_2_O is the major condensable species followed by S_2_ and S_4_. Although it’s still not clear exactly what is driving the overall temporal behavior of the albedo at 365 nm, a possible explanation is that it is driven by the variability in SO_2_ levels, in particular through the formation of some of the UV absorbers discussed in the literature (e.g. ref. ^26^) and/or from polysulfur and polysulfur oxide species. The implications of our model are that more complex sulfur compounds, beyond OCS, SO, SO_2_, SO_3_, and H_2_SO_4_, that are also produced by SO_2_ photochemistry could contribute to the absorption of UV in the enigmatic 320–400 nm range. Gas phase abundances of polysulfur and S_n_O by themselves are not capable of accounting for the NUV absorption but a combination of their condensed and gas phase forms (or in addition to FeCl_3_) might.

Our base modeling provides reasonable fits to available data, although the significance of these results is not entirely clear given the sparsity of data. The sporadic nature of observations of key species precludes making definitive conclusions regarding the major mechanisms involved in production of the enigmatic UV absorber and for understanding the photochemistry occurring in the upper atmosphere of Venus, especially given the large spatial and temporal variability of species that has been observed, e.g. by *Venus Express*. Rather, a more concentrated approach measuring a number of interacting species simultaneously would provide much needed input and hence greater confidence in our understanding of major chemical processes. In situ measurements using high resolution aerosol mass spectrometry (e.g. ref. ^27^) might be a way to identify the UV absorber on a future probe. Such measurements would be able to supply information not only identifying the major elements (S, O, C, Cl, N, and H) that may be present in the aerosol, but also their isotopic composition. Long term observations, such as those provided by *Venus Express* are crucial for integrating radiative, chemical and dynamical processes occurring on Venus.

Major uncertainties in the chemistry involve the reaction kinetics of SO and its dimers. Laboratory data on which the formation of SO dimers are calculated from ref. ^28^ and need to be updated. The photochemistry of the dimers and subsequent reactions of products also need to be examined in the laboratory. Likewise, kinetic data for many of the key reactions involving various S_n_ and S_n_O species are either lacking or need to be updated. In other words, the key uncertainties in both the laboratory and Venus data essentially remain the same as given by ref. ^8^. As noted by ref. ^29^, the terrestrial stratospheric sulphate layer (Junge layer), which is an important regulator of the Earth’s climate and the abundance of ozone, is highly similar to the upper haze layer on Venus. So, we think the results described here could be relevant for issues in stratospheric aerosol chemistry, namely, the evolution of stratospheric volcanic eruption clouds and geoengineering of the Earth’s climate.

## Methods

Our photochemical model of Venus is based on the Caltech/JPL KINETICS one-dimensional (1-D) model^8,9,21,24,29^. In brief, it is a 1-D diurnal-average steady-state model at the equator, extending in the vertical from 58 to 112 km with 0.2 km spacing from 58 to 78 km and 2 km spacing from 78 to 112 km. Vertical transport is parameterized via eddy diffusion. Radiative transfer processes in the model are the same as were described in ref. ^8^. Continuity equations are solved for 60 variable species. Mixing ratios of several key species are fixed throughout: CO_2_ = 0.965; N_2_ = 0.035; H_2_O = 1 ppm. The model contains ~500 thermal and photochemical reactions. Important chemical processes, including a high-altitude source of SO_2_ from H_2_SO_4_ photolysis are based mainly on ref. ^8^. The unusual high nighttime temperature at 95 km in ref. ^8^ is revised in this model. Constraint on H_2_SO_4_ is also applied in the model to fit the existing observations^16^. Photoabsorption cross sections and reaction rate coefficients are taken mainly from NASA and IUPAC compilations and lab studies^30–33^. The rate coefficient of the reaction S + CO + M → OCS + M, 3.0 × 10^-33^ exp(-1000/T) cm^6^s^-1^, is adopted from ref. ^34^. Lower boundary conditions are set for CO = 45 ppm, HCl = 0.4 ppm, SO_2_ = 3.0 ppm, OCS = 0.3 ppm, and NO = 5.5 ppb in our standard model. All other species produced within the model domain are allowed to flow downward with the eddy velocity (K/H). At the model’s upper boundary, a zero net flux condition is applied for CO, HCl and NO with respect to CO_2_, Cl + H, and N + O; for all other species a zero-flux condition is applied.

**Fig. 4:**
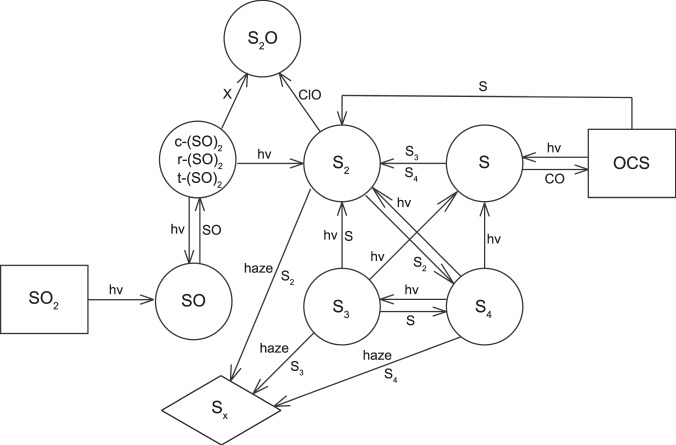
Major reactions in the model. Schematic diagram showing the main routes for producing S_x_ (shown in the diamond) from parent molecules, SO_2_ and OCS (shown in rectangles) in the model. Intermediate species are shown in the circles. Species needed for the reactions are written next to the arrows. X can be O, H, NO, S, SO, S_2_.

The sulfur cycle in our model is summarized in Fig. 4, based on an update of Figure A1 of ref. ^9^. The new reactions are summarized in Supplementary Table 1. The most important reactions in our chemical scheme are the formation of SO dimers, (SO)_2_, followed by their photolysis,

1 $${\mathrm{SO}} + {\mathrm{SO}} + {\mathrm{M}} \to c - \left( {{\mathrm{SO}}} \right)_2 + {\mathrm{M}}$$

2 $$\to {\mathrm{t}} - \left( {{\mathrm{SO}}} \right)_2 + {\mathrm{M}}$$

3 $$\to {\mathrm{cyclic}}\;\left( {{\mathrm{SO}}} \right)_2\ +\ {\mathrm{M}} \to {\mathrm{r}} - \left( {{\mathrm{SO}}} \right)_2$$

4 $${\mathrm{c}} - \left( {{\mathrm{SO}}} \right)_2\ +\ {\mathrm{hv}} \to \,{\mathrm{SO}} + {\mathrm{SO}}$$

5 $$\to {\mathrm{S}}_2 + {\mathrm{O}}_2$$

6 $${\mathrm{t}} - \left( {{\mathrm{SO}}} \right)_2\ +\ {\mathrm{hv}} \to {\mathrm{SO}} + {\mathrm{SO}}$$

7 $${\mathrm{r}} - \left( {{\mathrm{SO}}} \right)_2\ +\ {\mathrm{hv}} \to {\mathrm{S}} + {\mathrm{SO}}_2$$

where M is a third body, c-(SO)_2_, t-(SO)_2_ and r-(SO)_2_ are, respectively, the *cis*-, *trans*-, and trigonal- isomers of the SO dimer^4^. There is a fundamental difference between the formation of S_2_ by the above reactions and the previous mechanism^9^,

8 $${\mathrm{OCS}} + {\mathrm{hv}} \to {\mathrm{S}} + {\mathrm{CO}}$$

9 $${\mathrm{OCS}} + {\mathrm{S}} \to {\mathrm{S}}_2 + {\mathrm{CO}}$$

because this path for forming S_2_ may be aborted by the competing reaction,

10 $${\mathrm{S}} + {\mathrm{O}}_2 \to {\mathrm{SO}} + {\mathrm{O}}$$

thereby reducing the yield of S_2_ formation from OCS. By contrast, the pathways for S_2_ formation via c-(SO)_2_ and possibly r-(SO)_2_ do not involve the production of S atoms, and hence could not be short-circuited by O_2_.

Once S_2_ is produced, it readily self-propagates to form higher polymers of S via reactions such as:

11 $${\mathrm{S}}_2 + {\mathrm{S}}_2 + {\mathrm{M}} \to {\mathrm{S}}_4 + {\mathrm{M}}$$

12 $${\mathrm{S}}_4 + {\mathrm{S}}_2 + {\mathrm{M}} \to {\mathrm{S}}_6 + {\mathrm{M}}$$

13 $${\mathrm{S}}_4 + {\mathrm{S}}_4 + {\mathrm{M}} \to {\mathrm{S}}_8 + {\mathrm{M}}$$

S_n_, where *n* = 8 or larger, is lumped together as S_x_.

A number of abstraction reactions involving (SO)_2_

14 $$\left( {{\mathrm{SO}}} \right)_2 + {\mathrm{X}} \to {\mathrm{S}}_2{\mathrm{O}} + {\mathrm{XO}}\;{\mathrm{X}} = {\mathrm{O}},\;{\mathrm{H}},\;{\mathrm{NO}},\;{\mathrm{S}},\;{\mathrm{SO}},\;{\mathrm{S}}_2$$

producing S_2_O are also included. In addition, reactions involving Cl, such as

15 $${\mathrm{ClO}} + {\mathrm{S}}_2 \to {\mathrm{S}}_2{\mathrm{O}} + {\mathrm{Cl}}$$

and

16 $${\mathrm{ClS}} + {\mathrm{SO}} \to {\mathrm{S}}_2{\mathrm{O}} + {\mathrm{Cl}}$$

are also important sources of S_2_O. Once formed S_2_O can either photolyze or start polymerization via reactions, such as

17 $${\mathrm{S}}_2{\mathrm{O}} + {\mathrm{S}} + {\mathrm{M}} \to {\mathrm{S}}_3{\mathrm{O}} + {\mathrm{M}}$$

perhaps producing higher order S_n_O, in a manner similar to that for producing S_n_.

## Supplementary information

Supplementary Information

Peer Review File

## Data Availability

The model data and observational data that support the findings of this study are available from the corresponding author on reasonable request.

## Code availability

The code for the photochemical model written in Fortran and the post processing of the data written in Matlab is available from the corresponding author on reasonable request.
