# Supplementary material for: Sulfur monoxide dimer chemistry as a possible source of polysulfur in the upper atmosphere of Venus
Source: Nat Commun. 2021 Jan 8;12:175. doi: 10.1038/s41467-020-20451-2 (PMC7794339; doi:10.1038/s41467-020-20451-2)
Supplement: Supplementary file 1 — Supplementary Information [file 41467_2020_20451_MOESM1_ESM.pdf]

Supplementary Information: Sulfur monoxide dimer chemistry as a possible source of polysulfur in the upper atmosphere of Venus

Joseph P. Pinto[1], Jiazheng Li[2], Franklin P. Mills[3,4], Emmanuel Marcq[5], Daria Evdokimova[5,6], Denis Belyaev[6] and Yuk L. Yung[2,7]

[1]University of North Carolina at Chapel Hill, NC, USA
[2]Division of Geological and Planetary Science, California Institute of Technology, Pasadena, CA, USA
[3]Australian National University, Canberra, ACT, Australia
[4]Space Science Institute, Boulder, CO, USA
[5]LATMOS/CNRS/Sorbonne Université/UVSQ, France
[6]Space Research Institute of the Russian Academy of Sciences (IKI), Moscow, Russia
[7]Jet Propulsion Laboratory, California Institute of Technology, Pasadena, CA, USA

Corresponding author: Jiazheng Li (jiazheng@caltech.edu)

Supplementary Table 1. Reactions involving the SO dimer in the photochemical model.

Reactions	Photolysis Rate, J<br>Rate Coefficients, k*	References
**1. $SO + SO + M \rightarrow c\text{-}(SO)_2 + M$**	$k = 2.15\times10^{-31}$; 1.00×10^{-11}	a
**2. $SO + SO + M \rightarrow t\text{-}(SO)_2 + M$**	$k = 2.15\times10^{-31}$; 1.00×10^{-11}	a
**3. $SO + SO + M \rightarrow r\text{-}(SO)_2 + M$**	$k = 8.80\times10^{-33}$; 1.00×10^{-11}	a
**4. $c\text{-}(SO)_2 + h\nu \rightarrow SO + SO$**	$J = 9.30\times10^{-2}$	a
**5. $c\text{-}(SO)_2 + h\nu \rightarrow S_2 + O_2$**	$J = 9.30\times10^{-3}$	a
**6. $t\text{-}(SO)_2 + h\nu \rightarrow SO + SO$**	$J = 9.30\times10^{-2}$	a
**7. $r\text{-}(SO)_2 + h\nu \rightarrow S + SO_2$**	$J = 1.20\times10^{-1}$	a
**8. $O + c\text{-}(SO)_2 \rightarrow S_2O + O_2$**	$k = 3.00\times10^{-14}$	a
**9. $O + c\text{-}(SO)_2 \rightarrow SO + SO_2$**	$k = 3.00\times10^{-15}$	a
**10. $O + t\text{-}(SO)_2 \rightarrow S_2O + O_2$**	$k = 3.00\times10^{-14}$	a
**11. $O + t\text{-}(SO)_2 \rightarrow SO + SO_2$**	$k = 3.00\times10^{-15}$	a
**12. $H + c\text{-}(SO)_2 \rightarrow S_2O + OH$**	$k = 3.00\times10^{-14}$	b
**13. $H + t\text{-}(SO)_2 \rightarrow S_2O + OH$**	$k = 3.00\times10^{-14}$	b
**14. $NO + c\text{-}(SO)_2 \rightarrow S_2O + NO_2$**	$k = 3.00\times10^{-14}$	b
**15. $NO + t\text{-}(SO)_2 \rightarrow S_2O + NO_2$**	$k = 3.00\times10^{-14}$	b
**16. $SO + c\text{-}(SO)_2 \rightarrow S_2O + SO_2$**	$k = 3.00\times10^{-14}$	b
**17. $SO + t\text{-}(SO)_2 \rightarrow S_2O + SO_2$**	$k = 3.00\times10^{-14}$	a
**18. $S + c\text{-}(SO)_2 \rightarrow S_2O + SO$**	$k = 3.00\times10^{-14}$	a
**19. $S + t\text{-}(SO)_2 \rightarrow S_2O + SO$**	$k = 3.00\times10^{-14}$	a
**20. $S_2 + c\text{-}(SO)_2 \rightarrow 2S_2O$**	$k = 3.00\times10^{-14}$	a
**21. $S_2 + t\text{-}(SO)_2 \rightarrow 2S_2O$**	$k = 3.00\times10^{-14}$	a

*Low and high pressure rate coefficients given for SO dimer formation; J values given at 112 km, units are $cm^{-3}s^{-1}$ for J and $cm^3s^{-1}molec^{-1}$, $cm^6s^{-1}molec^{-2}$ for k. References: (a) ref. 1 and references therein; (b) estimated based on ref. 1.

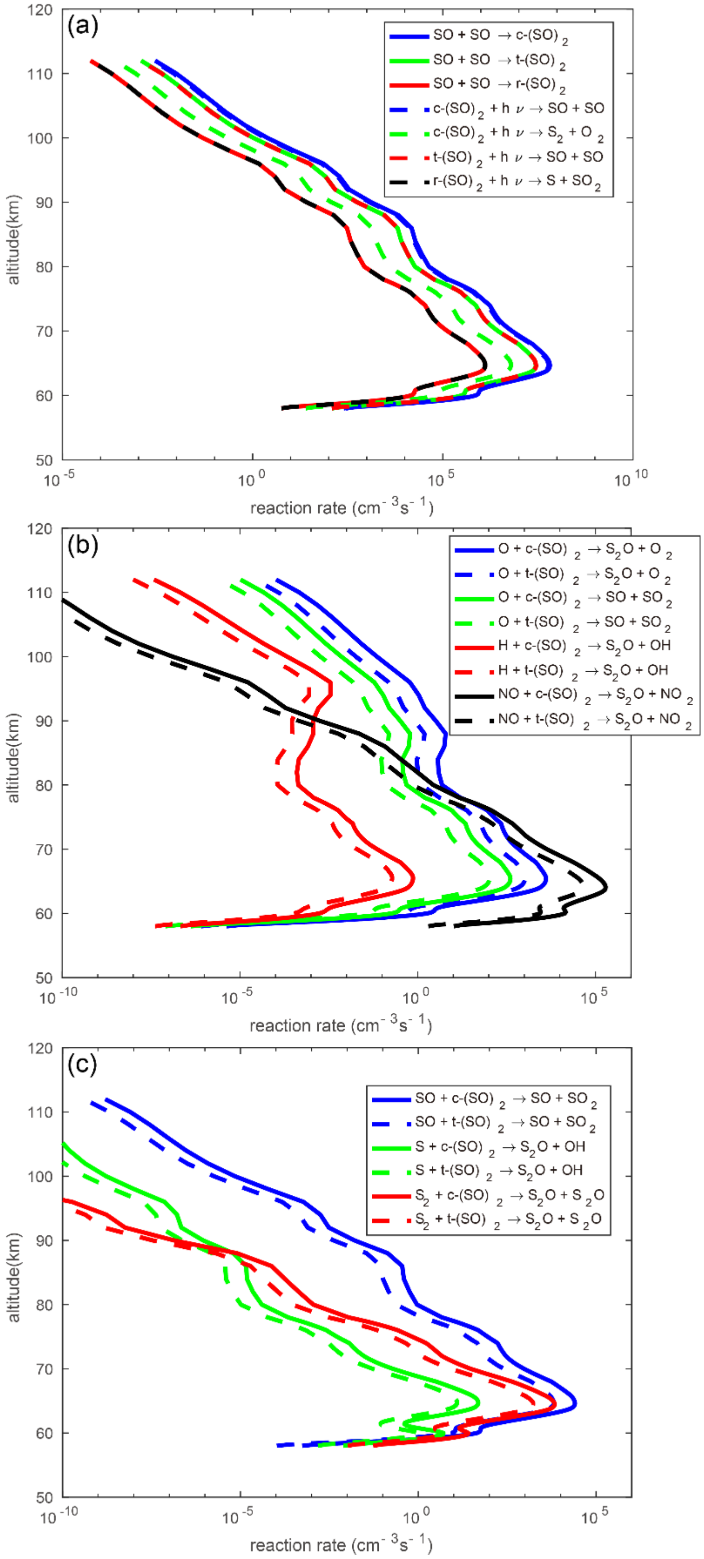

Supplementary Figure 1. Reaction rate profiles of the reactions in Table S1.

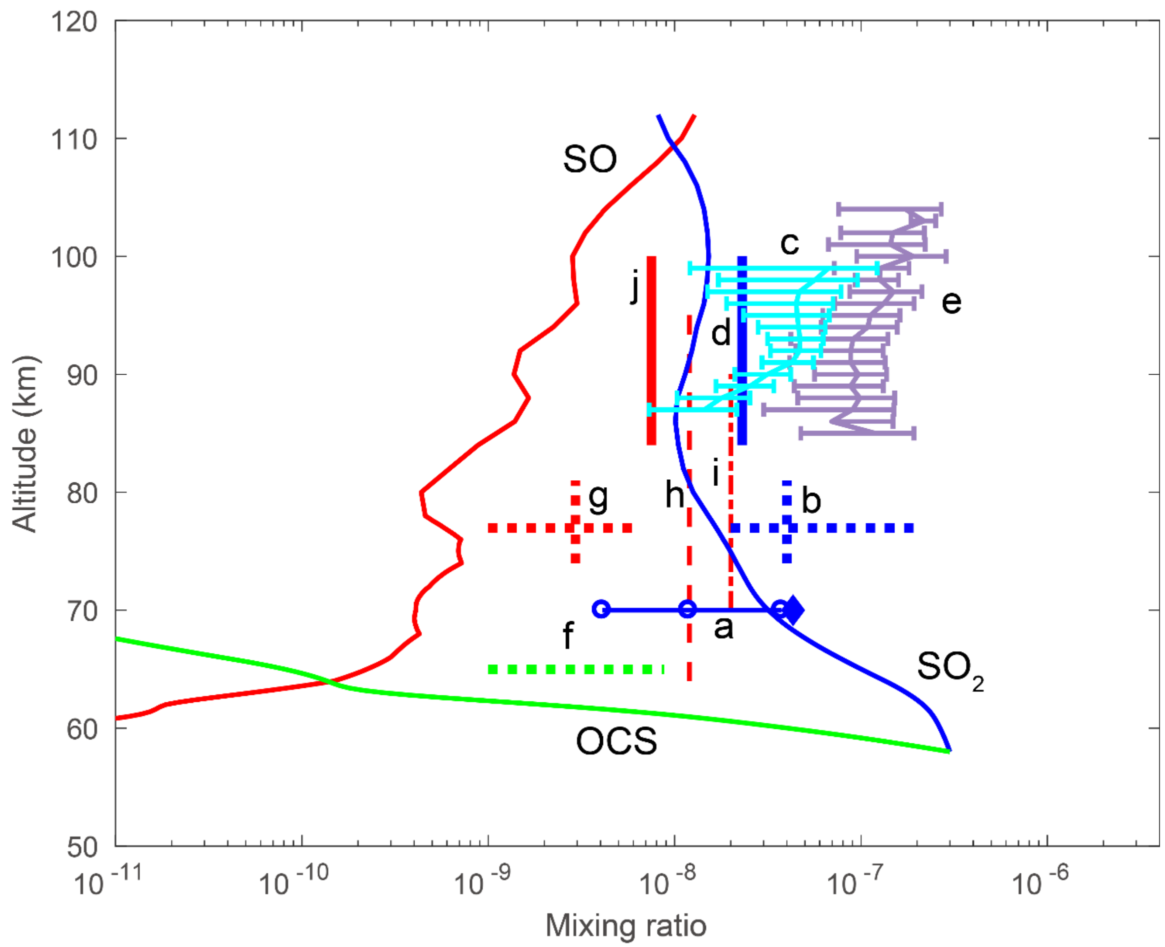

Supplementary Figure 2. Comparison of modeled profiles of SO_2, OCS and SO with observations for SO_2 = 0.3 ppm and OCS = 0.3 ppm at 58 km, the lower boundary of the model. Model profiles are shown as thin solid lines; (blue) SO_2, (green) OCS, (red) SO. Model SO_2 and SO profiles considering new H_2SO_4 cross sections from ref. 2 are shown as thin dotted lines. Model OCS profile using 100 ppm at 58 km shown as dotted line. Data sources: (a) SO_2, interquartile range from 2010 through 2014, diamond shows mean of distribution, ref. 3; (b) SO_2, ref. 4; (c) SO_2, ref. 5 solar occultation with 1-σ error bars; (d) SO_2, ref. 6; (e) SO_2, ref. 5 stellar occultation with 1-σ error bars; (f) OCS, ref. 7; (g) SO, ref. 4; (h) SO, ref. 8; (i) SO, ref. 9; (j) SO, ref. 6.

References

1. Zhang, X., Liang, M. C., Mills, F. P. et al (2012). Sulfur chemistry in the middle atmosphere of Venus. *Icarus*, *217*, 714-739.

2. Farahani, S., Frandsen, B. N., Kjaergaard, H. G., et al. (2019). Simulated Electronic Absorption Spectra of Sulfur-Containing Molecules Present in Earth's Atmosphere. *The Journal of Physical Chemistry A*, *123*, 6605-6617.

3. Marcq, E., Jessup, K. L., Baggio, L. et al. (2020). Climatology of SO_2 and UV absorber at Venus' cloud top from SPICAV-UV nadir dataset. *Icarus*, *335*, 113368.

4. Jessup, K. L., Marcq, E., Mills, F. et al. (2015). Coordinated Hubble Space Telescope and Venus Express observations of Venus' upper cloud deck. *Icarus*, *258*, 309-336.

5. Belyaev, D. A., Evdokimova, D. G., Montmessin, F., et al. (2017). Night side distribution of SO2 content in Venus' upper mesosphere. *Icarus*, *294*, 58-71.

6. Sandor, B. J., Clancy, R. T., Moriarty-Schieven, G., et al. (2010). Sulfur chemistry in the Venus mesosphere from SO2 and SO microwave spectra. *Icarus*, *208*, 49-60.

7. Krasnopolsky, V. A. (2010). Spatially-resolved high-resolution spectroscopy of Venus 2. Variations of HDO, OCS, and SO2 at the cloud tops. *Icarus*, *209*, 314-322.

8. Na, C. Y., Esposito, L. W., McClintock, W. E. et al. (1994). Sulfur dioxide in the atmosphere of Venus: II. Modeling results. *Icarus*, *112*, 389-395.

9. Na, C. Y., Esposito, L. W., & Skinner, T. E. (1990). International ultraviolet explorer observation of Venus SO_2 and SO. *Journal of Geophysical Research: Atmospheres*, *95*, 7485-7491.
